# Neuron-specific enolase promotes stem cell-like characteristics of small-cell lung cancer by downregulating NBL1 and activating the BMP2/Smad/ID1 pathway

**DOI:** 10.1038/s41389-022-00396-5

**Published:** 2022-04-29

**Authors:** Lin Lu, Zhiqiang Zha, Peiling Zhang, Peipei Wang, Xia Liu, Xisheng Fang, Chengyin Weng, Baoxiu Li, Haibo Mao, Lina Wang, Mingmei Guan, Yong Wu, Zhixiang Xu, Zhongqiu Liu, Guolong Liu

**Affiliations:** 1 Department of Medical Oncology, Guangzhou First People’s Hospital, School of Medicine, South China University of Technology, 510180 Guangzhou, Guangdong China; 2 Department of Medical Oncology, Guangzhou First People’s Hospital, Guangzhou Medical University, 510180 Guangzhou, Guangdong China; 3 Division of Hematology and Oncology, Comprehensive Cancer Center, the University of Alabama at Birmingham, Birmingham, AL 35233 USA; 4 International Institute for Translational Chinese Medicine, Guangzhou University of Chinese Medicine, 510006 Guangzhou, Guangdong China

**Keywords:** Cancer stem cells, Small-cell lung cancer

## Abstract

Little is known about the biological functions of neuron-specific enolase (NSE) as a specific biomarker for small-cell lung cancer (SCLC). Herein, we elucidate the effect and mechanism of NSE on SCLC stem cell-like characteristics. Upregulated NSE expression was observed in spheroid cells. The gain-of-function and loss-of-function approaches demonstrated that modulation of NSE positively regulated cell proliferation, drug resistance, spherical clone formation, tumor growth, and stem cell-like characteristics of SCLC cells. Mechanistic studies revealed that NSE might downregulate the expression of neuroblastoma suppressor of tumorigenicity 1 (NBL1) by interacting with NBL1, thereby attenuating the competitive inhibitory effect of NBL1 on BMP2 and enhancing the interaction between BMP2 and BMPR1A; this, in turn, may activate the BMP2/Smad/ID1 pathway and promote SCLC stem cell-like characteristics. Moreover, overexpression of NBL1or knockdown of BMP2 rescued the NSE-induced stem cell-like characteristics. In clinical specimens, NSE expression was positively associated with ALDH1A1 expression and negatively correlated with NBL1 expression. High NSE and ALDH1A1 expressions and low NBL1 expression were correlated with poor prognosis in patients with SCLC. In summary, our study demonstrated that NSE promoted stem cell-like characteristics of SCLC via NBL1 and the activation of the BMP2/Smad/ID1 pathway.

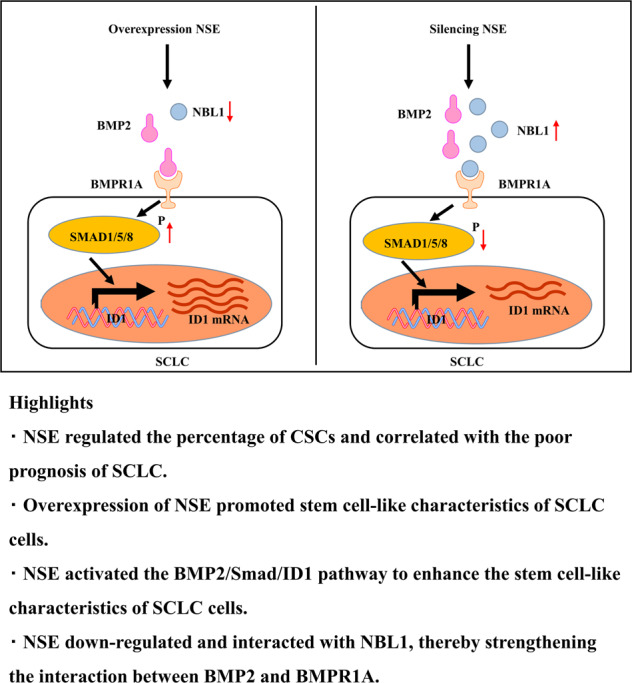

## Introduction

Small-cell lung cancer (SCLC) represents 10–25% of lung cancers; however, it is the most malignant lung cancer associated with high mortality [1–3]. SCLC is frequently related to widespread metastases in the early stages, and the 5-year survival rate for patients with extensive-stage (ES) SCLC is less than 5% [4–6]. The application of targeted therapies and immune checkpoint inhibitors are not very effective for most patients with SCLC [5, 6]. Accumulating evidence indicates that cancer stem cells (CSCs) are responsible for tumor progression and relapse [7–10]. CSCs have also been found to be closely related to tumor progression in SCLC [11]. Therefore, the identification of novel targets and insights into the molecular events involved in the stem cell-like characteristics of SCLC are urgently needed to help us better understand the properties of SCLC and develop more effective therapeutic strategies for patients with SCLC.

Neuron-specific enolase (NSE), encoded by the ENO2 gene, is explicitly expressed by neurons and peripheral neuroendocrine cells. NSE has only been used as a specific and reliable biomarker for SCLC [12–15]. The concentrations of serum NSE have been correlated with tumor differentiation, treatment response, relapse, and metastasis in patients [16–19]. We previously found that silencing NSE upregulated the expression of E-cadherin, which promotes stem cell-like characteristics [20, 21]. Thus, we aimed to determine whether NSE could provide SCLC with stem cell-like features to promote tumor progression.

In this study, the results demonstrated that NSE activated the BMP2/Smad/ID1 signaling pathway by interacting with NBL1 and downregulating it, promoting the stem cell-like characteristics of SCLC cells. Our study revealed a novel role and mechanism by which NSE regulates the stem cell-like characteristics and malignant behavior of SCLC cells.

## Materials and methods

### Characteristics of patients and ethical statement

In all, 82 cases with SCLC were included in this study. None of the patients had received any antitumor therapy before collecting the tissue samples. Only patients with SCLC with complete follow-up data were included in this study. All human tissues were obtained with informed consent, and the study was approved by the Research Ethics Committee of Guangzhou First People’s Hospital, South China University of Technology.

### Mice and ethical statement

All animal experiments conformed to animal protocols approved by the Animal Care and Use Committee of the South China University of Technology. Female BALB/c nude mice 6–8 weeks of age were purchased from Hunan SJA Laboratory Animal Co. Ltd. and housed under specific pathogen-free conditions. Five mice were randomly assigned to each group for experiments.

### Cell line and cell culture

The human SCLC cell lines H209, H446, and H69 were purchased from Genetimes ExCell Technology, Inc. and cultured in a complete medium containing RMPI-1640 supplemented with 10% FBS. Cells were identified by the STR (short tandem repeat) authentication and free of mycoplasma infection.

### Flow cytometry

The enrichment of ALDH^high^ CSCs was detected using the ALDEFLUOR kit as previously described [22, 23]. Briefly, 1 × 10^6^ cells were incubated with ALDEFLUOR substrate. Tumor cells incubated with the ALDEFLUOR substrate and DEAB were used as negative controls. The enrichment of ALDH^high^ CSCs was analyzed using a flow cytometer (Beckman Coulter) and sorted on a flow cell sorter (BD FACSAria SORP).

### Real-time quantitative PCR

Total RNA was isolated from the indicated SCLC cells using TRIzol reagent and was reverse transcribed to cDNA. The resulting cDNA was subjected to qRT-PCR. Quantitative real-time PCR was performed using iQ SYBR Green Supermix and an iCycler Real-time PCR Detection System (Bio-Rad). The primer sequences used are listed in Supplementary Table 1. The relative expression of the target genes was normalized to the transcription level of 18S rRNA.

### Western blot

Tumor cells were collected and lysed using RIPA lysis buffer to generate proteins, as previously described [24]. After quantification using the Bradford assay kit, equal amounts of proteins were fractionated and transferred onto polyvinylidene fluoride membranes. The membranes were incubated with the following antibodies: NSE (1:2000, Abcam), GFP (1:2000, Proteintech), Flag (1:1000, Proteintech), OCT4 (1:1000, CST), Nanog (1:2000, CST), SOX2 (1:1000, CST), β-actin (1:5000, Proteintech), BMP2 (1:1000, Proteintech), Smad1 (1:2000, Proteintech), pSmad1/5/8 (1:1000, CST), and ID1 (1:500, Proteintech). After incubation with HRP-conjugated anti-rabbit or anti-mouse secondary antibodies (1:10,000; Bio-Rad), the signal was detected using an enhanced chemiluminescence (ECL) kit.

### Plasmids and established stable cells

The CDS sequence of the human NSE or NBL1 gene was subcloned into a pSIN-3× FLAG lentiviral vector. NSE was inserted into the pSIN-GFP lentiviral vector. All shRNAs and their corresponding negative control (NTC) in the PLKO vector were purchased from Sigma-Aldrich. The shRNA-targeting sequences are listed in Supplementary Table 2. Viruses expressing shRNA or CDS sequences of human NSE or NBL1 were produced in HEK293T cells. A lentivirus plasmid containing scrambled sequences was used to construct the negative control (NTC) cells for shRNA knockdown. Viruses produced from the empty plasmids were used to transfect cells to construct the empty vector (EV) control for gene overexpression cells. We used two viruses in a mix to infect cells with two different lentiviral vectors. Polybrene was added to improve the infection efficiency, and puromycin was selected to establish stable cells.

### Cell proliferation

Cells in the exponential growth phase were collected and seeded in 96-well plates at a density of 2 × 10^3^ per well. Next, 10% (volume/volume) of CCK8 (GLPBIO) was added to the culture medium and incubated for 2 h. Cell viability was assessed using the CCK8 assay, which was monitored by measuring the absorbance at 450 nm using a multifunction microporous plate inspection and analysis system (BiotekCytation5).

### Tumorigenicity

The tumorigenicity of SCLC cells was assessed using a nude mouse xenograft model. A total of 5 × 10^5^ cells were inoculated subcutaneously into the right flank of BALB/c nude mice (6–8 weeks). The tumor volumes were recorded twice per week by investigators without blinding. Tumor volume was calculated as follows: tumor volume = 1/2(length × width^2^). After sacrificing the mice, tumor masses were harvested and weighed.

### Sphere formation

The sphere-formation capability of the SCLC cells was assessed as previously described [25]. Briefly, 500 cells were cultured in six-well ultra-low attachment surface plates (Costar) in serum-free culture medium, which consisted of DMEM/F12 medium, 1 × B27, 20 ng/ml bFGF, 20 ng/ml EGF, and 10 ng/ml insulin. The culture medium was replaced with fresh medium every alternate day. After 14 d, representative graphs and sphere counts were obtained.

### Colony formation

Colony formation was used to detect cell proliferation capability. Tumor cells at a density of 500 cells were seeded in a six-well plate and cultured for 14 days. Colonies were fixed with 4% formaldehyde and stained with 0.5% crystal violet. Colonies were counted under a microscope (≥50 cells per colony).

### Chemoresistance

Isolated cells were seeded into 96-well culture plates at 5 × 10^3^ cells/well for one day and then treated with increasing concentrations of cisplatin (0–20 μg/ml) for 24 h. Cell viability was measured using the methyl-thiazol-tetrazolium assay. The absorbance at 490 nm was measured using a Multi-Mode Reader Cytation5 (Biotek, Winooski, VT, USA) to observe the chemosensitivity of cancer cells to cisplatin.

### RNA sequencing

Total RNA was isolated from the negative control (H69-NTC) and NSE-knockdown SCLC cells (H69-shNSE) using the TRIzol reagent and was sent to BGI Shenzhen for sequencing. In this study, all samples were measured using the DNBSEQ platform, and each sample produced an average of 21.77 M data. The reads were aligned to the human genome, GRCh38.p11(hg38). This project used SOAPnuke software for filtering, HISAT software for reference genome alignment, and Bowtie2 for comparing clean reads to reference gene sequences. RSEM was used to calculate the expression levels of the genes and transcripts. All unigenes were aligned to the AnimalTFDB2.0 database, and differential expression analysis was performed using PossionDis, with a false discovery rate (FDR) ≤ 0.001 and |Log2Ration | ≥1 [26]. KEGG annotation was performed using R software’s “phyper” function for enrichment analysis based on differentially expressed genes.

### Immunohistochemistry

Immunohistochemistry was routinely conducted as previously described [27, 28]. After antigen retrieval, the slides were incubated with the following primary antibodies: NSE (1:100; Abcam), rabbit anti-ALDH1A1 polyclonal antibody (1:200; Abcam), and NBL1 (1:200; Affinity) overnight at 4 °C. Immunohistochemical staining was performed using diaminobenzidine (DAB). After counterstaining with hematoxylin, the sections were dehydrated and sealed. Staining scores were assessed by two different individuals (ZQZ and PLZ). The percentage and intensity of staining were evaluated and multiplied to obtain the final scores. The principle of immunohistochemical scoring is mainly based on staining intensity (0–3 points) and staining area (0–3 points). Finally, the product is the total score. Dyeing intensity was as follows: 0 points for no dyeing, 1 point for light yellow, 2 points for yellow or brown, 3 points for brown or tan; the stained area was as follows: 0 points for ≤5%, 1 point for 5–25%, 2 points for 25–50%, and 3 points for ≥50%. An immunohistochemical score of ≤3 indicated a low expression group, and a score >3 indicated a high expression group [29].

### Double-immunofluorescence labeling

For double-immunofluorescence staining, the slides were routinely baked, dewaxed, and hydrated as previously described [27, 28]. After antigen retrieval using hot citrate buffer (pH = 6), the slides were incubated with a blocking reagent (goat serum, Boster) for 1 h at room temperature. SCLC sections were incubated with the following primary antibodies: mouse anti-NSE monoclonal antibody (1:150; Proteintech) and rabbit anti-NBL1 polyclonal antibody (1:200; Affinity) overnight at 4 °C. The sections were then incubated with secondary antibodies for 2 h at room temperature after thorough washing, followed by staining with DAPI staining solution (Beyotime) for 15 min. The secondary antibodies used were Dylight488, goat anti-mouse IgG (H + L) (Earthox) and Dylight649, and goat anti-rabbit IgG (H + L) (Earthox). Sections were mounted using an Antifade reagent (Applygen) for fluorescence microscopy.

### Extreme limiting dilutions analysis (ELDA)

The single-cell suspension was plated at a low density in a 96-well ultra-low attachment plate. We set up ten repetition groups, then incubated cells in a 37 °C, 5% CO_2_ humidified incubator for 14 days. By day 14, the number of wells containing the tumor cell spheres was counted. Stemness frequencies were calculated using the ELDA website (http://bioinf.wehi.edu.au/software/elda/). The default confidence interval was set at 0.95. The resulting plots were exported.

### Co-immunoprecipitation (Co-IP)

Cells were lysed using a low-salt IP buffer containing one × cocktail solution. One piece of cocktail (05056489001, Roche) was dissolved in 1 ml water to obtain 50 × cocktail solutions. Supernatants were obtained by high-speed centrifugation. The supernatant was then added to 20 µL protein A/G-conjugated beads to wash the sample, quantify the protein, obtain 3 µg protein, and dilute to 1000 µL. The samples from each group were equally divided into IgG and input groups. The corresponding antibody (IgG 1 µg, GFP 1 µg, or FLAG 1 µg) was added to the supernatant and incubated overnight. The supernatants were then added to protein A/G-conjugated beads and incubated for 2 h. Finally, the beads were collected, washed, and analyzed by western blotting.

### Cancer cell line encyclopedia (CCLE) database analysis

CCLE (https://portals.broadinstitute.org/ccle/home) is an open-access database containing large-scale deep-sequencing information from 947 human cancer cell lines [30]. The mRNA expression of ENO2 (encoding NSE) and NBL1 in SCLC cell lines were extracted for further analysis. Spearman analysis was used to assess the linear relationship between ENO2 and NBL1, and *P* < 0.05 was considered statistically significant (*P < 0.05; **P ≤ 0.01; ***P ≤ 0.001).

### Statistical analysis

Quantitative values are expressed as mean ± standard deviation (SD). All data were statistically analyzed using GraphPad Prism 7. 0. Unpaired Student’s *t* test (two cohorts) or one-way analysis (>two cohorts) was used to determine the differences between experimental groups. Univariate and multivariate survival analyses were performed using the likelihood ratio test of stratified Cox proportional risk regression. Survival analysis was performed using the log-rank test. Statistical significance was defined as a two-tailed *P* value <0.05.

## Results

### NSE is highly expressed in sphere cells and regulates the percentage of CSCs

**Fig. 1:**
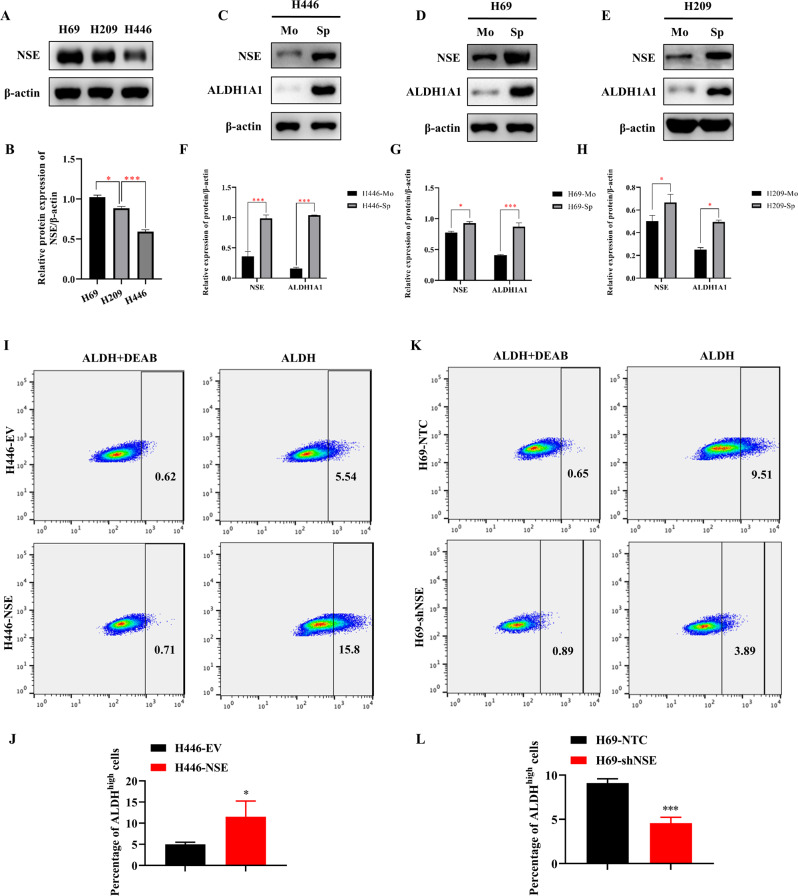
NSE is highly expressed in sphere cells and regulates the percentage of CSCs. **A** Western blot detects the expression level of NSE in different SCLC cell lines, and the relative protein levels (NSE/β-actin) were quantified as the column graph (**B**). **C**–**E** The expression levels of NSE and ALDH1A1 in spherical cells were significantly increased (**C**: H446 cell; **D**: H69 cell; **E**: H209 cell). **F**–**H** All western blot images were quantified by the ImageJ software to estimate the relative protein expression (protein /β-actin) (**F**: H446 cell; **G**: H69 cell; **H**: H209 cell). **I** NSE overexpression enriched the percentage of the ALDH^high^ CSC population. NSE overexpression in H446 cells (H446-NSE), negative control cells (H446-EV). **J** The bar charts represent the proportion of the ALDH^high^ CSC population. **K** Knockdown of NSE diminished the ALDH^high^ CSC population in SCLC cells. They silenced NSE in H69 cells (H69-shNSE) negative control cells (H69-NTC). **L** The bar charts represent the percentage of the ALDH^high^ CSC population. Results are shown as mean ± SD of independent experiments, and each experiment was repeated thrice.

To characterize the role of NSE in SCLC stem cell-like characteristics, we first detected NSE expression in various SCLC cells. As shown in Fig. 1A, B, the expression level of NSE was highest in H69 cells, followed by H209 cells, while NSE was lowest in H446 cells. Therefore, H446 cells were virally transfected with NSE to generate a stable cell line overexpressing NSE (Supplementary Fig. S1A, B, E). NSE expression was stably knocked down by transfecting H69 cells with a lentivirus carrying an NSE shRNA (Supplementary Fig. S1C–E). The ALDH1A1 expression was upregulated in spherical cells, implying an enhanced stemness trait of spherical cells (Fig. 1C–H). Moreover, we found that NSE expression was significantly increased in spherical cells, suggesting a positive relationship between NSE expression and tumor stemness (Fig. 1C–H). NSE overexpression increased the percentage of ALDH^high^ cells (Fig. 1I, J, *P* = 0.039). Silencing NSE resulted in a significant reduction in the rate of ALDH^high^ CSCs (Fig. 1K, L, *P* < 0.001).

### NSE regulates SCLC tumor growth in vitro and in vivo

**Fig. 2:**
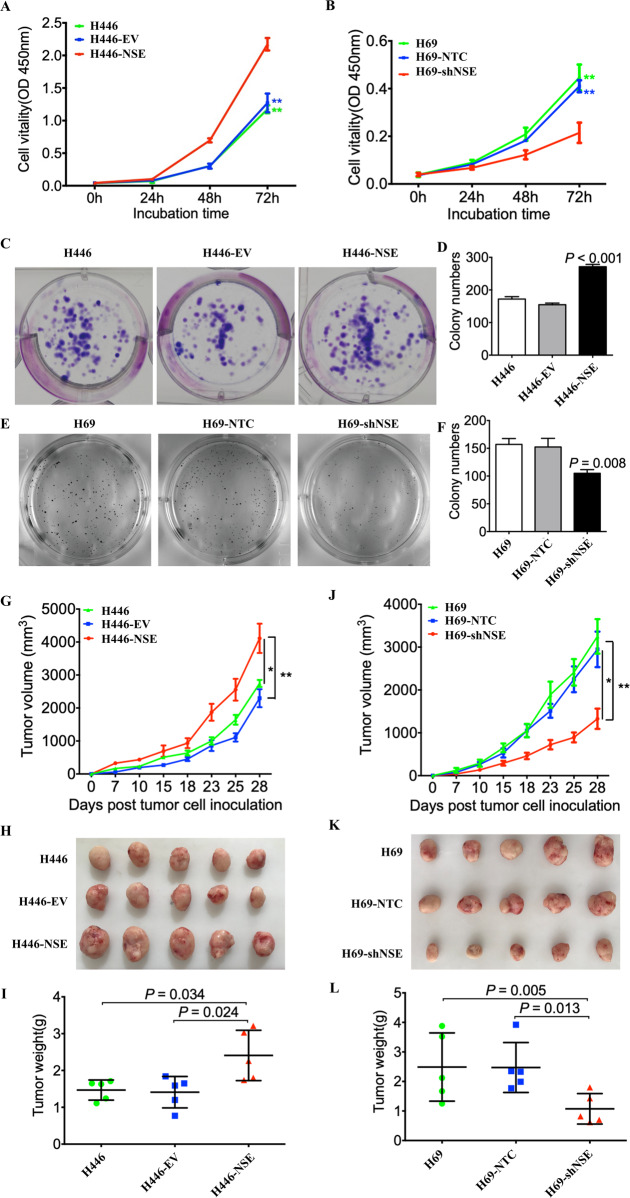
NSE promotes SCLC tumor growth in vitro and in vivo. The cell proliferation capability was assessed using the CCK8 assay. **A** Overexpression of NSE promoted the cell proliferation capability of H446 cells. **B** Knockdown of NSE repressed cell proliferation of H69 cells. **C** NSE overexpression promoted the colony-formation capability of SCLC cells. **D** Colonies were stained with crystal violet, and the number of colonies was counted and shown in a column diagram. Error bars represent the mean ± SD of three independent experiments. **E** Silencing of NSE inhibited the colony-formation capability of SCLC cells. **F** The number of colonies was counted and is shown in a column diagram. Error bars represent mean ± SD from three independent experiments. **G**–**I** Overexpression of NSE promotes tumor growth in vivo. **G** The tumor growth curves were compared between NSE overexpression cells, empty vector cells, and untransduced cells. **H** The images showed the macroscopic appearance of tumors resected from NSE overexpression or control tumors. **I** The image showed the statistical analyses of the tumor weights. **J**–**L** Silencing NSE inhibited the tumor-initiating capability of SCLC cells. **J** The tumor growth curves are shown. **K** The images show the macroscopic appearance of tumors resected from NSE silencing cells or negative control tumors. **L** The image shows the statistical analyses of the tumor weights. Data are represented as mean ± SD for three experiments. *P < 0.05; **P < 0.01.

We investigated whether NSE was a critical regulator of SCLC cell growth. CCK8 assay demonstrated that NSE overexpression led to a remarkable increase in cell proliferation (Fig. 2A, *P* < 0.01). The proliferation ability of SCLC cells was significantly reduced in shNSE cells compared with that in scrambled control cells (Fig. 2B and Supplementary Fig. S2F, *P* < 0.01). The colony-formation ability of SCLC cells was enhanced by NSE overexpression (Fig. 2C, D, *P* < 0.001) and was significantly inhibited after NSE knockdown (Fig. 2E, F, *P* = 0.008).

Next, we studied the in vivo oncogenic effects of NSE using a xenograft model in nude mice. NSE-overexpressing H446 cells exhibited enhanced efficiency in forming large tumors. The average volume and weight of tumors in the NSE-overexpression group were markedly higher than those in the control group (Fig. 2G–I). NSE knockdown significantly inhibited tumor growth (Fig. 2J–L). These data indicate that NSE promotes the growth of SCLC cells.

### NSE modulates stem cell-like characteristics of SCLC cells

**Fig. 3:**
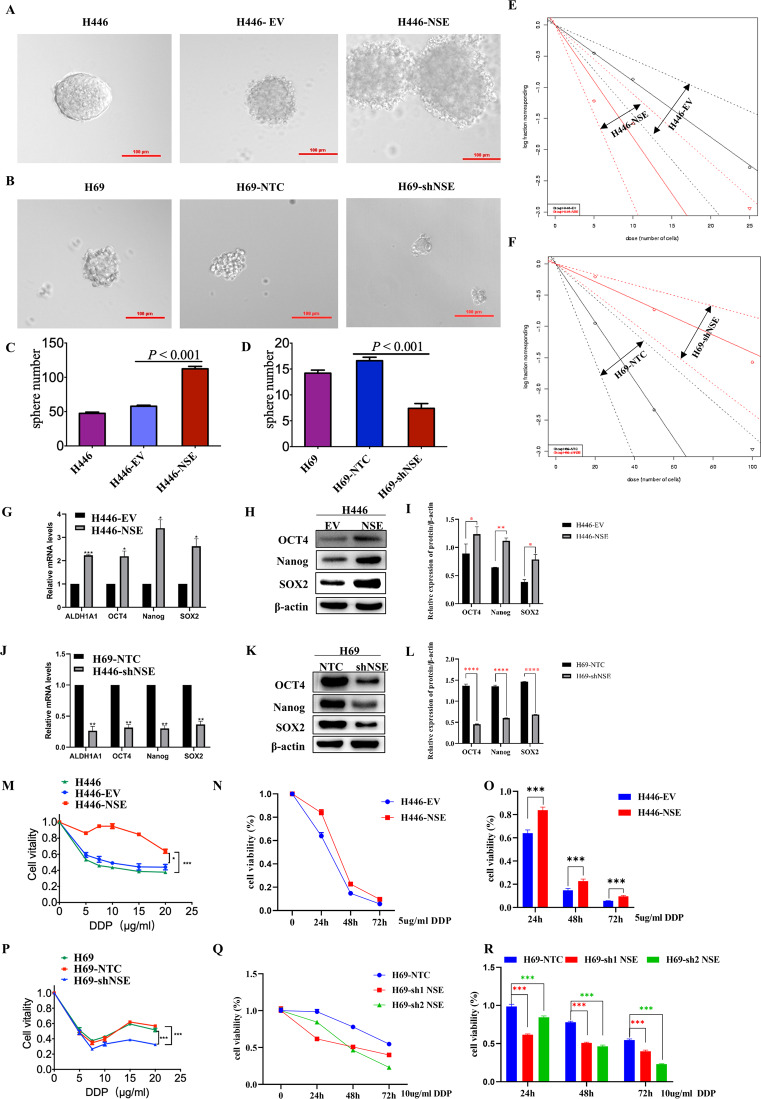
NSE modulates the stem cell-like characteristics of SCLC cells. The self-renewal capability of SCLC cells was determined by assessing the sphere formation. **A** Representative morphology of the sphere formed by NSE-overexpressing cells (original magnification ×20, scale bar 100 μm). **B** Representative images of sphere formation were displayed after 14 days of culture in NSE-knockdown cells (original magnification ×20, scale bar 100 μm). **C** The bar charts represented the percentage of spheroid cells of NSE-overexpressing cells compared to the control cells. **D** The bar charts represented the percentage of spheroid cells of NSE-knockdown cells compared to the control cells. **E**, **F** The stemness property of H446 cells (**E**) and H69 cells (**F**) was evaluated by the Extreme Limiting Dilutions Analysis website. The mRNA (**G**) and protein levels (**H**) of stemness-associated genes upon NSE overexpression were detected. **I** The relative protein expression (protein/β-actin) was analyzed by ImageJ software and quantified in a column graph. NSE knockdown inhibited the expression of CSC-related genes at the mRNA level (**J**) and protein level (**K**). **L** All western blot images were analyzed by ImageJ software, and the relative protein expression (protein/β-actin) was quantified in a column graph. SCLC cells in indicated groups were treated with cisplatin, and the cell viability was measured using the CCK8 assay. **M**–**O** Compared with EV cells, cells overexpressing NSE showed a dose-dependent decrease (**M**) and time-dependent decrease (**N**) in the sensitivity to cisplatin. **O** The proportion of alive cells in SCLC cells was also shown as column graphs. **P**–**R** NSE-knockdown cells were more sensitive to cisplatin than negative control cells with a dose-dependent decrease (**P**) and time-dependent decrease (**Q**). **R** The percentage of alive cells in SCLC cells is also shown as column graphs. Data are represented as mean ± SD for three experiments. *P < 0.05; **P < 0.01; ***P < 0.001.

CSCs are characterized by self-renewal, the expression of stemness markers, and chemoresistance [31]. First, overexpression of NSE resulted in the formation of a greater number and increased size of spheres than those of spheres from H446-empty vector (EV) cells (Fig. 3A, C, *P* < 0.001). The spheres formed by NSE-silenced cells were smaller than H69-negative control (NTC) cells (Fig. 3B, D, *P* < 0.001). In addition, we found that silencing NSE inhibited the formation of spheres in H209 cells (Supplementary Fig. S2C, D, *P* < 0.05). Extreme limiting dilution analysis was used to evaluate the stemness properties of SCLC cells. The results demonstrated that compared with the control group, NSE-overexpressing H446 cells had a more robust ability to give rise to clones while silencing NSE inhibited the capacity of producing spheres (Fig. 3E, F and Supplementary Fig. S2E, *P* < 0.05). The mRNA expression levels of ALDH1A1, OCT4, NANOG, and SOX2 were upregulated in NSE-overexpressing H446 cells (Fig. 3G). In addition, protein levels of stemness markers were elevated in NSE-overexpression H446 cells, including OCT4, Nanog, and SOX2 (Fig. 3H, I). The expression of these CSC-related markers was downregulated in NSE-knockdown H69 cells (Fig. 3J–L). In addition, we found that silencing NSE downregulated the expression of CSC-related markers in H209 cells (Supplementary Fig. S2A, B). Cell viability indicated the effect of NSE on chemoresistance upon cisplatin treatment. As shown in Fig. 3M–O, the viability of H446 cells was enhanced by NSE overexpression under cisplatin treatment. NSE-knockdown H69 and H209 cells demonstrated greater cisplatin vulnerability than control cells (Fig. 3P–R and Supplementary Fig. S2G, H, *P* < 0.05). Taken together, these data indicate that NSE induces CSC-like properties in SCLC cells.

### NSE enhances stem cell-like characteristics of SCLC cells by activating the BMP2/Smad/ID1 pathway

**Fig. 4:**
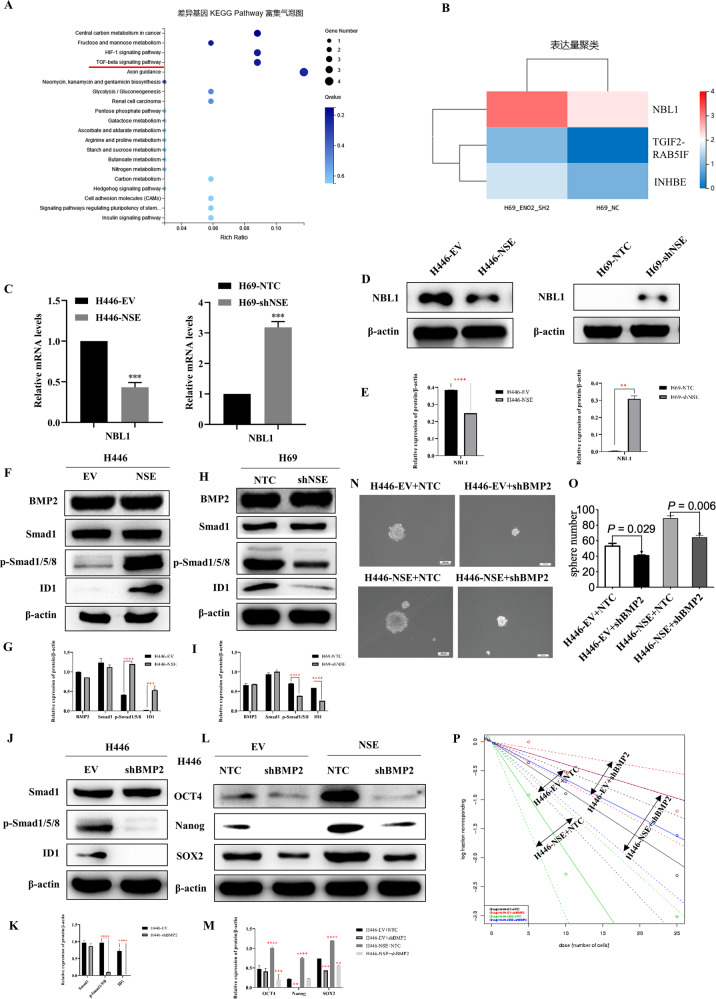
NSE enhances the stem cell-like characteristics of SCLC cells through activating the BMP2/Smad/ID1 pathway. **A** KEGG functional enrichment analysis revealed significant enrichment in TGF-βsignaling in H69-shNSE cells compared to the control cells. **B** The heatmap showed the differential expression of genes involved in the TGF-β signaling pathway between H69-shNSE and control cells. **C** Relative NBL1 mRNA levels in SCLC cells with overexpression or knockdown NSE. **D** Western blot images showing NBL1 expression was regulated by overexpression or knockdown NSE. **E** The relative expression of NBL1 in different groups was shown as histograms. **F** Western blot images showing total protein content of Smad1, pSmad1/5/8, ID1, and the housekeeping gene β-actin in H446 cells transduced with the empty vector (EV) and vector overexpressing NSE. **G** All western blot images were analyzed by ImageJ software, and the relative protein expression (protein/β-actin), including total Smad1, pSmad1/5/8, ID1, was quantified in a column graph. **H** Western blot images showing total protein content of Smad1, pSmad1/5/8, ID1, and β-actin in H69 cells transduced with the scrambled shRNA (NTC) and shNSE. **I** The relative protein expression, including total Smad1, pSmad1/5/8, ID1, was normalized to β-actin and quantified as a column graph. **J** Western blot images showing protein levels of Smad1, pSmad1/5/8, ID1, and β-actin in H446 cells with silencing BMP2 compared to the control cells. **K** The column graph represented the relative protein expression, including total Smad1, pSmad1/5/8, ID1, with β-actin as normalization. **L** The protein levels of OCT4, Nanog, and SOX2 were analyzed using western blot in H446 cells infected with NSE or shBMP2 plasmid compared to the control cells. **M** The histogram showed the relative protein expression, including OCT4, Nanog, and SOX2, normalizing to β-actin. **N** The stemness capability of NSE-overexpression cells was evaluated by the sphere-formation analysis (original magnification ×20, scale bar 100 μm). **O** Quantitative analysis of spheroid cells. Data are presented as mean values ± SD for three independent experiments. **P** The stemness capability of NSE-overexpression cells was analyzed by the Extreme Limiting Dilutions Analysis website.

RNA sequencing was performed to detect differentially expressed genes between H69-shNSE and H69-NTC cells to determine the underlying mechanism by which NSE modulates stem-like properties of SCLCs. There were 70 significant and differentially genes in H69-shNSE and control cells. The KEGG pathway analysis revealed that NSE might significantly act on the TGF-β signaling pathway (Fig. 4A). We show the differential expression of genes involved in the TGF-β signaling pathway as the heatmap, including NBL1, TGIF2-RAB5IF, and INHBE, and NBL1 was the most significantly different gene (Fig. 4B). Many studies have suggested that the TGF-β pathway can promote tumor proliferation, invasion, metastasis, tissue fibrosis, angiogenesis, immune function, and acquisition of stemness traits in lung cancer [32–34]. As NBL1 can serve as an antagonist of the TGF-β pathway by competitively inhibiting BMP [35], we used real-time quantitative PCR and western blot experiments to detect the expression of NBL1. Overexpression of NSE in H446 cells decreased NBL1 expression, whereas silencing NSE in H69 cells upregulated NBL1 mRNA and protein levels (Fig. 4C–E).

NBL1 specifically inhibits BMP2 and BMP4, and the paralogs of NBL1, Grem2, has a higher affinity for BMP2 than for BMP4 [35, 36]. Hence, we used western blotting to detect the expression of BMP2 and its downstream target genes Smad1, pho-Smad1/5/8, and ID1, all of which are involved in the TGF-β pathway [37–39]. As shown in Fig. 4F, G, NSE overexpression resulted in upregulation of pho-Smad1/5/8 and ID1. NSE silencing downregulated the expression of pho-Smad1/5/8 and ID1 (Fig. 4H, I). Meanwhile, modulation of NSE did not regulate the expression of BMP2 and total Smad1/5/8. We further inhibited the TGF-β pathway by silencing BMP2 expression (Fig. 4J, K). shBMP2 was virally transfected into H446 cells to generate a stable cell line with BMP2 knockdown (Supplementary Fig. S1F–H). Moreover, silencing of BMP2 in NSE-overexpressing H446 cells eliminated NSE-mediated stemness-related gene expression (Fig. 4L, M) and sphere formation in SCLC cells (Fig. 4N–P). In summary, our results show that the BMP2/Smad/ID1 pathway is activated and plays a vital role in NSE-induced stem cell-like characteristics of SCLC cells.

### NSE downregulates and interacts with NBL1 in SCLC cells

**Fig. 5:**
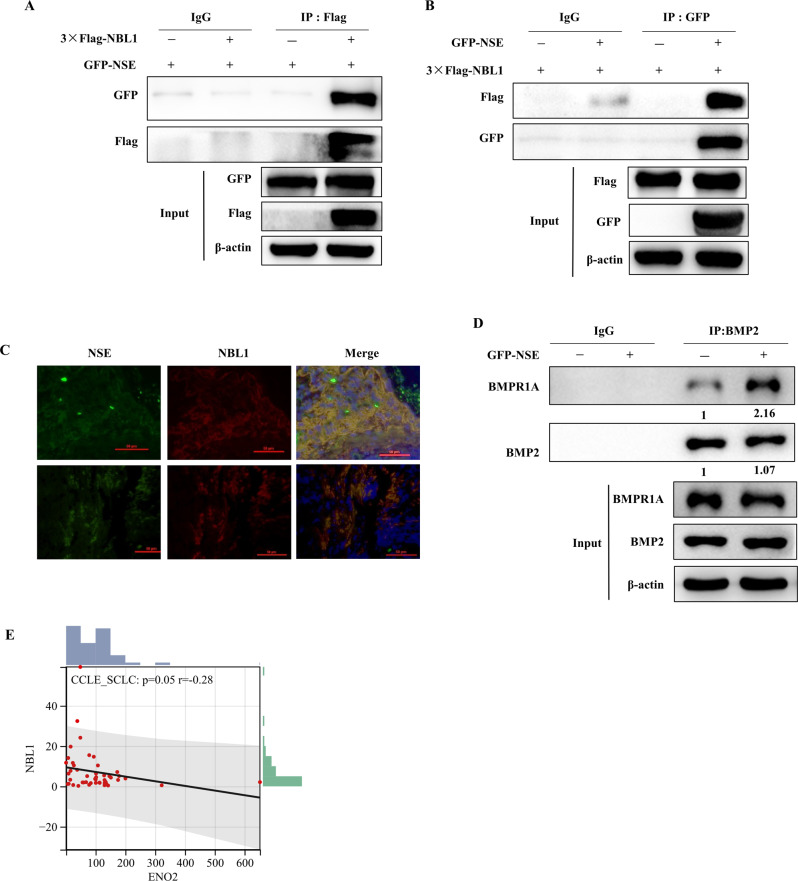
NSE downregulates and interacts with NBL1 in SCLC cells. **A**, **B** Western blot images showed the interaction between NSE and NBL1 by Co-IP experiment. **C** The double-immunofluorescence labeling experiment detected NSE and NBL1 and displayed co-location in clinical samples (original magnification ×40, scale bar 50 μm). **D** Western blot images show the interaction of BMP2 and BMPR1A enhanced after overexpression of NSE. **E** The photo shows a significant and negative correlation between ENO2 (encode NSE) and NBL1 in mRNA level using the CCLE database.

We further explored the molecular mechanism by which NSE activates the BMP2/Smad/ID1 pathway. NBL1 acts as an antagonist of the TGF-β pathway by competitively inhibiting BMP2 [35]. Therefore, we performed co-IP experiments to examine whether there was an interaction between NSE and NBL1. These results confirmed that NSE and NBL1 could interact, explaining the mechanism by which NSE regulates NBL1 (Fig. 5A, B). Immunofluorescence results also showed that NSE and NBL1 were co-localized (Fig. 5C). Moreover, NSE overexpression enhanced the interaction between BMP2 and BMPR1A (Fig. 5D). Furthermore, we analyzed the correlation between NSE and NBL1 expression in 47 SCLC cell lines in the CCLE database, which demonstrated a statistically significant negative correlation (Fig. 5E).

In summary, our research showed that NSE might reduce the competitive inhibitory effect of NBL1 on BMP2 by interacting with NBL1, thereby enhancing the interaction between BMP2 and BMPR1A and activating the BMP2/Smad/ID1 pathway.

### NBL1 participates in the NSE-mediated modulation of stem cell-like characteristics of SCLC

**Fig. 6:**
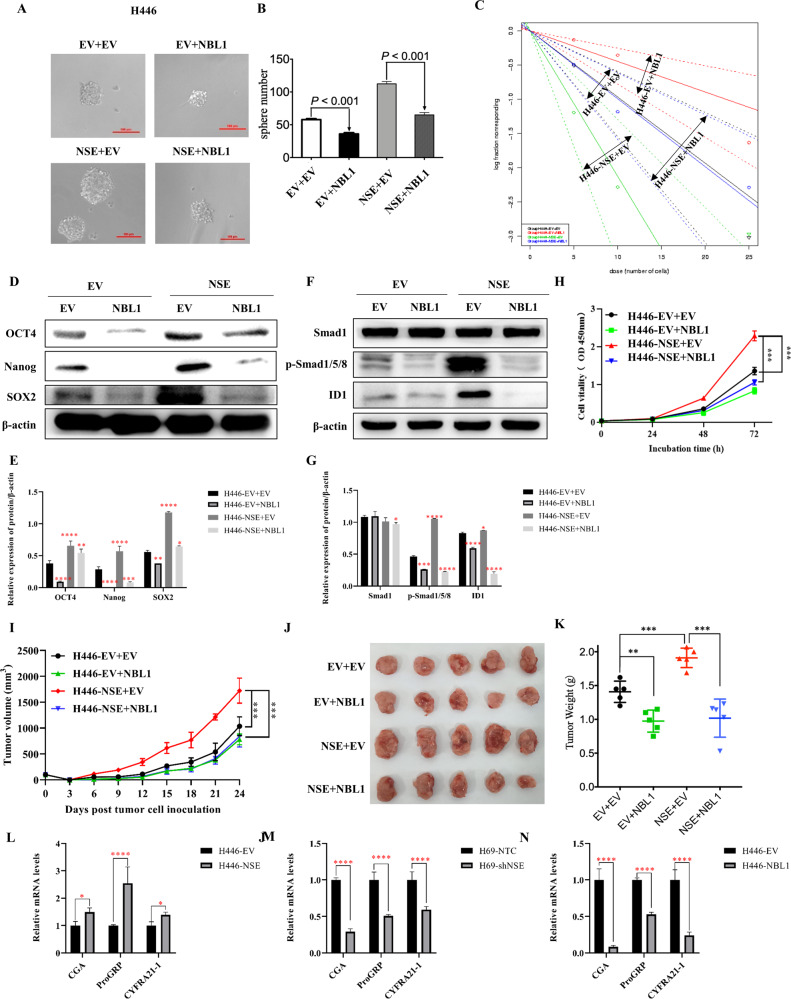
NBL1 participates in the NSE-mediated stem cell-like characteristics of SCLC. **A** Sphere-formation capability of NSE-overexpressing SCLC cells followed by NBL1 overexpression (original magnification ×20, scale bar 100 μm). **B** Quantitative analysis of spheroid cells in various subgroups. Data are presented as mean values ± SD for three independent experiments. **C** The stemness property of NSE-overexpressing SCLC cells followed by NBL1 overexpression was detected by the Extreme Limiting Dilutions Analysis. **D** The protein levels of multiple stemness-related genes were analyzed in H446 cells with overexpressed NSE, with or without NBL1 overexpression compared to the control cells, and the relative protein expression (protein/β-actin) was quantified in a column graph (**E**). **F** The total protein content of Smad1, pSmad1/5/8, ID1, and β-actin in H446 cells with overexpressed NSE, with or without NBL1 overexpression compared to the control cells. **G** The bar chart showed the protein expression of total Smad1, pSmad1/5/8, and ID1, which was normalized to β-actin. **H** Cell proliferation was assessed using CCK8 assay. The pro-proliferation ability of NSE overexpression was counteracted by NBL1 overexpression. The tumorigenicity of NSE-overexpressing cells was diminished by NBL1 overexpression. **I** Tumor volumes were assessed every 3 days until 24 days after tumor cell injection. **J** Tumor tissues derived from nude mice inoculated tumor cells were displayed. **K** Tumor weights were examined on day 24 and displayed as a histogram. **L** Relative changes in the mRNA levels of CgA, ProGRP, and CYFRA21-1 in H446 cells overexpressing NSE. **M** The mRNA levels of CgA, ProGRP, and CYFRA21-1 in H69 cells with silenced NSE were detected by qRT-PCR. **N** Bar charts showing significant differential expression of CgA, ProGRP, and CYFRA21-1 in H446 cells with elevated NBL1 expression. Data are represented as mean ± SD for three experiments. The level of significance is indicated by *P < 0.05; **P < 0.01; ***P < 0.001.

Next, we investigated whether the NSE-mediated stem cell-like characteristics were dependent on NBL1. H446 cells were transfected with the NSE and NBL1 plasmids. NBL1 was stably overexpressed by transfecting H446 cells with a lentivirus carrying NBL1 and was detected using qRT-PCR and western blot analysis (Supplementary Fig. S1I–K). The efficiency of H446 cells transfected with NSE and NBL1 plasmids was determined by western blotting (Supplementary Fig. S1L–N). As shown in Fig. 6A–C, NSE overexpression enhanced tumor sphere-forming capacity, which was counteracted by NBL1 overexpression. Moreover, upregulation of CSC-related markers (OCT4, NANOG, and SOX2) induced by NSE overexpression was compensated by NBL1 increase (Fig. 6D, E). Overexpression of NBL1 abolished the activation of the BMP2/Smad/ID1 pathway by the overexpression of NSE (Fig. 6F, G). Furthermore, elevated NBL1 counteracted the promotion of NSE overexpression on SCLC cell proliferation in vitro (Fig. 6H) and tumor growth in vivo (Fig. 6I–K). In addition, we also detected several specific neuroendocrine properties of SCLC, including pro-gastrin-releasing peptide (ProGRP), chromogranin A (CGA), and the cytokeratin marker CYFRA21-1 [40, 41]. The results showed that increased NSE expression led to the upregulation of ProGRP, CGA, and CYFRA21-1 while silencing of NSE downregulated the expression of these markers (Fig. 6L, M). A similar experiment was performed to overexpress NBL1. The results showed that NBL1 significantly inhibited CgA, ProGRP, and CYFRA21-1 expression (Fig. 6N). The above studies suggest that NSE expression increases tumorigenicity and that NBL1 suppresses tumorigenicity. Our results indicate that NSE promotes cell growth and maintains the stem cell-like characteristics of SCLC cells via NBL1.

### Clinical significance of NSE, ALDH1A1, and NBL1 in patients with SCLC

**Fig. 7:**
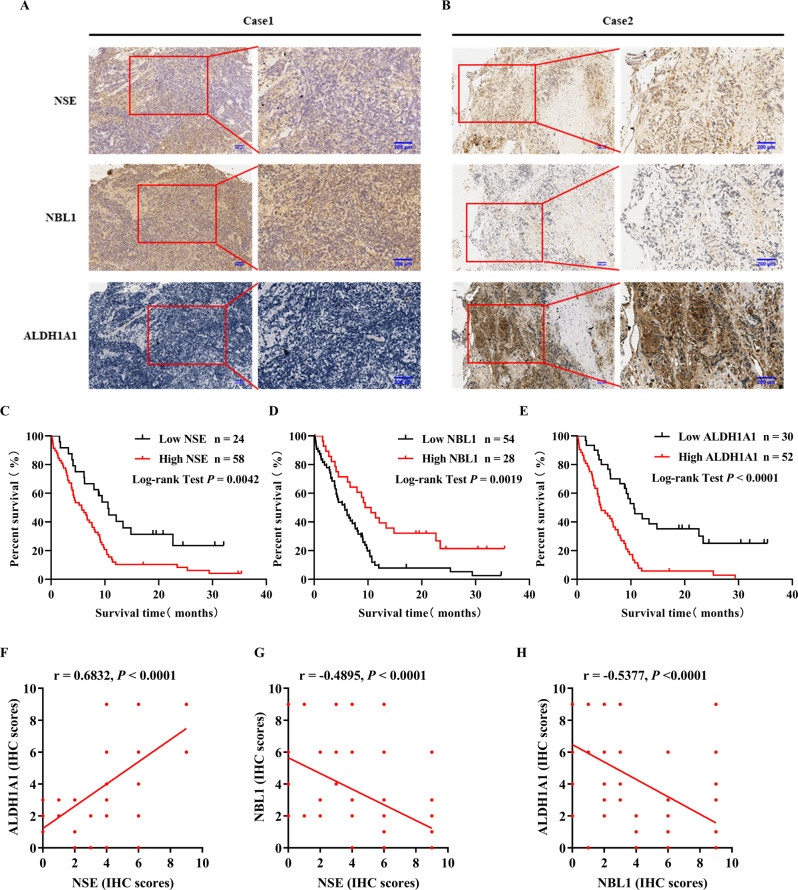
The clinical significance of NSE, NBL1, and ALDH1A1 expression in patients with SCLC. In all, 82 cases of SCLC tissues were stained with NSE, NBL1, and ALDH1A1 in serial paraffin-embedded tissue blocks. Representative microscopy images of paired NSE, NBL1, and ALDH1A1 staining are shown at 10X and 20X magnifications, scale bar 200 μm. **A** Images on the left represent one case with high NSE staining, low NBL1 staining, and high ALDH1A1 staining. **B** Images on the right demonstrate a representative case with low NSE expression, high NBL1 expression, and low ALDH1A1 expression. Kaplan–Meier survival analysis was performed to determine the role of NSE (**C**), NBL1 (**D**), and ALDH1A1 (**E**) expression in predicting the overall survival of patients with SCLC. Spearman’s correlation coefficient was used to assess the correlation between NSE, NBL1, and ALDH1A1. **F** Positive correlation between NSE and ALDH1A1 expression. **G** Negative correlation between NSE and NBL1 expression. **H** Negative correlation between NBL1 expression and ALDH1A1 expression.

Patients with SCLC were divided into two groups based on the median NSE serum concentration (25 ng/ml). Clinicopathological parameters and NSE distributions are summarized in Table 1. We found that a high NSE serum concentration (>25 ng/ml) was related to lymph node metastasis (*P* = 0.036), distant metastasis (*P* < 0.001), and higher TNM stage (*P* = 0.004). Moreover, univariate Cox regression analysis revealed that NSE, N stage, and M stage correlated with the prognosis of patients with SCLC (Supplementary Fig. S1O, *P* < 0.05). However, multivariate analysis showed that only the M stage could be an independent prognostic parameter (Supplementary Fig. S1P, *P* < 0.05). Immunohistochemistry was used to detect the expression of NSE, NBL1, and ALDH1A1 in serial paraffin-embedded SCLC tissues. High expression rates of NSE, NBL1, and ALDH1A1 were 70.7%, 34.1%, and 63.4%, respectively. Figure 7A, B shows representative microscopic images of two patients with SCLC. Kaplan–Meier survival analysis demonstrated that high NSE and ALDH1A1 expression levels contributed to a shorter overall survival time. In contrast, low NBL1 expression predicted poor overall survival for patients with SCLC (Fig. 7C for NSE, Fig. 7D for NBL1, Fig. 7E for ALDH1A1). Moreover, there was a positive correlation between NSE and ALDH1A1 expression in tumor tissues (Fig. 7F). There was a negative correlation between NBL1 and NSE expression (Fig. 7G). The expression of NBL1 was also negatively correlated with ALDH1A1 expression (Fig. 7H).

**Table 1:** The correlations between NSE serum concentrations and clinicopathological parameters of SCLC patients.

Clinicopathological factors	*n*	NSE		χ^2^	*P*
		>25 ng/ml	≤25 ng/ml		
Age					
<60	26	20	6	0.308	0.579
≥60	56	46	10		
Gender					
Female	6	3	3	3.831	0.05
Male	76	63	13		
Tumor depth					
≤3 cm	4	2	2	2.489	0.115
>3 cm	78	64	14		
Lymph metastasis					
N0	3	1	2	4.409	0.036*
N1	79	65	14		
Distant metastasis					
M0	26	14	12	17.206	<0.001*
M1	56	52	4		
TNM stage					
I/II	2	0	2	8.456	0.004*
III/IV	80	66	14		

*P < 0.05.

## Discussion

NSE has long been used as a tumor biomarker for diagnosis and to monitor treatment efficacy and relapse in patients with SCLC [14, 15]. However, the biological functions of NSE in SCLC remain largely unknown. Our previous study demonstrated that NSE modulated the proliferation and migration ability and downregulated the expression of E-cadherin; therefore, more studies are needed to explore the effect of NSE on SCLC stem cell-like characteristics [42].

CSCs represent a small population of tumor cells and are generally accepted as the source of tumor progression and relapse [43, 44]. CSCs are characterized by self-renewal, chemoresistance, sphericity, tumorigenicity, and specific markers. We found that NSE and ALDH1A1 were highly expressed in the SCLC sphere cells. Overexpression of NSE promotes cell proliferation and tumorigenicity in SCLC cells. Moreover, NSE enhanced the acquisition of stem cell-like characteristics. Overexpression of NSE increased the percentage of ALDH^high^ CSCs, enhanced sphere-formation capability, upregulated the expression of stemness-associated genes, and repressed chemosensitivity. In contrast, the knockdown of NSE diminished these effects. To the best of our knowledge, this is the first study to demonstrate the role of NSE in stem cell-like characteristics of SCLC cells.

Since there is a lack of evidence contributing to the underlying mechanisms of NSE in SCLC stem cell-like characteristics, we revealed that NSE enhances stem cell-like characteristics of SCLC cells by interacting and decreasing NBL1 and strengthening the interaction between BMP2 and BMPR1A to activate the BMP2/Smad/ID1 pathway. NBL1 is an antagonist of BMP2, also known as neuroblastoma (DAN), a tumor suppressor that plays essential roles in numerous biological functions [45]. Reduced NBL1 expression increases cell proliferation, migration, and invasion [46]. Notably, the effects of the BMP signaling pathway on the maintenance of CSC-like traits remain controversial [47–49]. Moreover, NBL1 overexpression or knockdown of BMP2 abrogated NSE-promoted CSC-like properties in SCLC cells. Here, we conclude that NSE promotes and maintains stem cell-like characteristics of SCLC cells by downregulating and interacting with NBL1, thereby activating the BMP2/Smad/ID1 pathway.

Intriguingly, through clinical data analysis, a high serum concentration of NSE was found to be related to a high tumor stage in patients with SCLC. In addition, immunohistochemical analysis of the expression levels of NSE, NBL1, and ALDH1A1 in the tumor tissues of patients with SCLC showed that the expression level of NSE was positively correlated with that of ALDH1A1 and negatively correlated with that of NBL1. Our study assessed the relationships between NSE, NBL1, and ALDH1A1 to help us identify reliable and accurate markers to reveal the stem cell-like characteristics of SCLC.

In conclusion, we demonstrated that NSE activates the BMP2/Smad/ID1 pathway by downregulating and interacting with NBL1 to promote stem cell-like characteristics of SCLC cells. This study provides novel insights into the function of NSE and the mechanism by which NSE regulates stem cell-like characteristics and malignant behaviors of SCLC. We believe that these findings may be beneficial for the development of SCLC therapies.

## Supplementary information

Supplementary_material

Supplementary Figure 1

Supplementary Figure 2

## Data Availability

All data in our study are available from the corresponding authors upon reasonable request.
