# Supplementary material for: Neuron-specific enolase promotes stem cell-like characteristics of small-cell lung cancer by downregulating NBL1 and activating the BMP2/Smad/ID1 pathway
Source: Oncogenesis. 2022 Apr 29;11(1):21. doi: 10.1038/s41389-022-00396-5 (PMC9054797; doi:10.1038/s41389-022-00396-5)
Supplement: Supplementary file 1 — Supplementary_material [file 41389_2022_396_MOESM1_ESM.docx]

**Supplementary Figure S1** (A) Overexpression of NSE in H446 cells was verified by western blot, and the relative NSE expression normalized to β-actin was shown as a bar chart (B). (C) Knockdown of NSE in H69 cells was confirmed by western blot, and the relative NSE level (NSE/β-actin) was quantified as a column (D). (E) Overexpression or knockdown of NSE in SCLC cells verified by qRT-PCR. (F) Knockdown of BMP2 in H446 cells confirmed by western blot. (G) Relative BMP2 expression (BMP2/β-actin) in H446-shBMP2 cells was quantified as a histogram. (H) The knockdown efficiency of BMP2 in H446 cells was also verified by qRT-PCR. Overexpression of NBL1 in H446 cells was detected by western blot (I), and the histogram represented the relative NBL1 expression (J). (K) The overexpression efficiency of NBL1 in H446 cells was also verified by qRT-PCR. The transfection efficiency of NSE or NBL1 in SCLC cells was analyzed by western blot (L-M), and the relative expression of proteins was shown as a bar graph (N). The relationship between serum NSE concentration and the prognosis of SCLC patients was evaluated by univariate (O) and multivariate (P) COX regression analysis.

**Supplementary Figure S2** (A) The protein levels of NSE and stemness-related genes on silencing NSE H209 cells by western blot, and quantified as relative protein expression of interested protein/β-actin (B). (C) Representative images of sphere formation were observed using an inverted microscope after 14 days of culture in silencing NSE H209 cells (Original magnification 20X, scale bar 100 μm). (D) The bar charts represented the percentage of spheroid cells in H209- shNSE cells compared to the control cells. (E) The stemness property of H209 cells was evaluated by the Extreme Limiting Dilutions Analysis. (F) The cell proliferation capabilities of H209 cells were assessed using CCK8 assay. (G, H) Silencing NSE H209 cells were more sensitive to cisplatin than negative control cells with a dose-dependent (G) and time-dependent decrease (H).

Supplementary Table 1 Primers used in qRT-PCR

| Gene name | Primer sequence |
| --- | --- |
| NSE | (Forward) AGCCTCTACGGGCATCTATGA |
|  | (Reverse) TTCTCAGTCCCATCCAACTCC |
| OCT4 | (Forward) CTGGGTTGATCCTCGGACCT |
|  | (Reverse) CCATCGGAGTTGCTCTCCA |
| Nanog | (Forward) AATGGTGTGACGCAGGGATG |
|  | (Reverse) TGCACCAGGTCTGAGTGTTC |
| Sox2 | (Forward) TGGACAGTTACGCGCACAT |
|  | (Reverse) CGAGTAGGACATGCTGTAGGT |
| ALDH1A1 | (Forward) GGAGGAAACCCTGCCTCTTTT |
|  | (Reverse) TTGGAAGATAGGGCCTGCAC |
| NBL1 | (Forward) TGTTCCCAGATAAGAGTGCCT |
|  | (Reverse) GCAGGAGTCACAGTGAACCAG |
| BMP2 | (Forward) ACCCGCTGTCTTCTAGCGT |
|  | (Reverse) TTTCAGGCCGAACATGCTGAG |
| 18s | (Forward) CGGCGACGACCCATTCGAAC |
|  | (Reverse) GAATCGAACCCTGATTCCCCGTC |

Supplementary Table 2 shRNAs used in PLKO vector

| Gene name | shRNA sequence |
| --- | --- |
| Sh1 NSE | (Forward)CCGGCAAGGGAGTCATCAAGGACAACTCGAGTTGTCCTTGATGACTCCCTTGTTTTTG |
|  | (Reverse)AATTCAAAAACAAGGGAGTCATCAAGGACAACTCGAGTTGTCCTTGATGACTCCCTTG |
| Sh2 NSE | (Forward)CCGGGCGTTACTTAGGCAAAGGTGTCTCGAGACACCTTTGCCTAAGTAACGCTTTTTG |
|  | (Reverse)AATTCAAAAAGCGTTACTTAGGCAAAGGTGTCTCGAGACACCTTTGCCTAAGTAACGC |
| Sh1 BMP2 | (Forward)CCGGCAAGATGCTTTAGGAAACAATCTCGAGATTGTTTCCTAAAGCATCTTGTTTTTG |
|  | (Reverse)AATTCAAAAACAAGATGCTTTAGGAAACAATCTCGAGATTGTTTCCTAAAGCATCTTG |
| Sh2 BMP2 | (Forward)CCGGCCGGAGATTCTTCTTTAATTTCTCGAGAAATTAAAGAAGAATCTCCGGTTTTTG |
|  | (Reverse)AATTCAAAAACCGGAGATTCTTCTTTAATTTCTCGAGAAATTAAAGAAGAATCTCCGG |
