# Supplementary figures and images for: Neuron-specific enolase promotes stem cell-like characteristics of small-cell lung cancer by downregulating NBL1 and activating the BMP2/Smad/ID1 pathway
Source: Oncogenesis. 2022 Apr 29;11(1):21. doi: 10.1038/s41389-022-00396-5 (PMC9054797; doi:10.1038/s41389-022-00396-5)

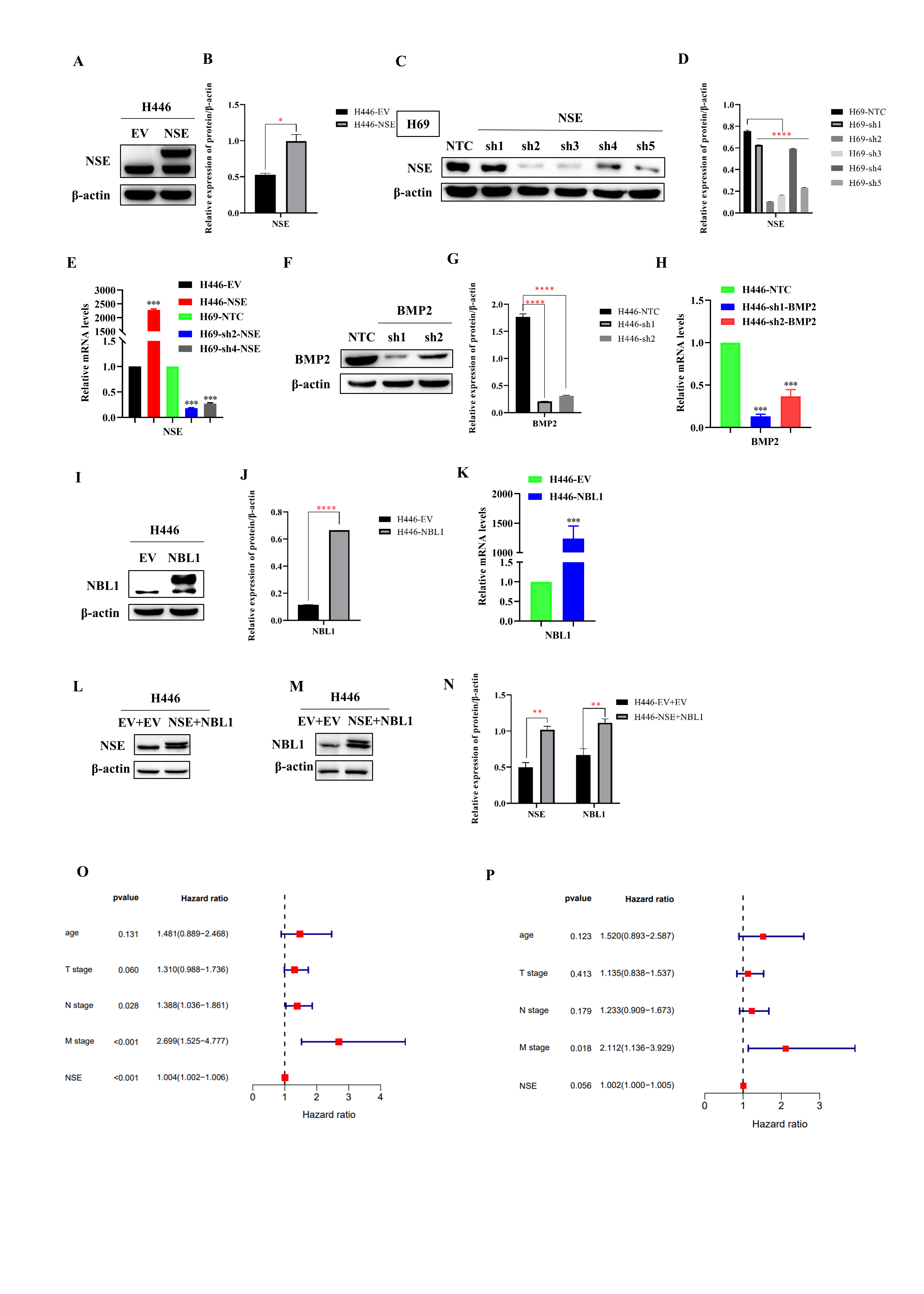

Supplement: Supplementary file 2 — Supplementary Figure 1 [file 41389_2022_396_MOESM2_ESM.tif]

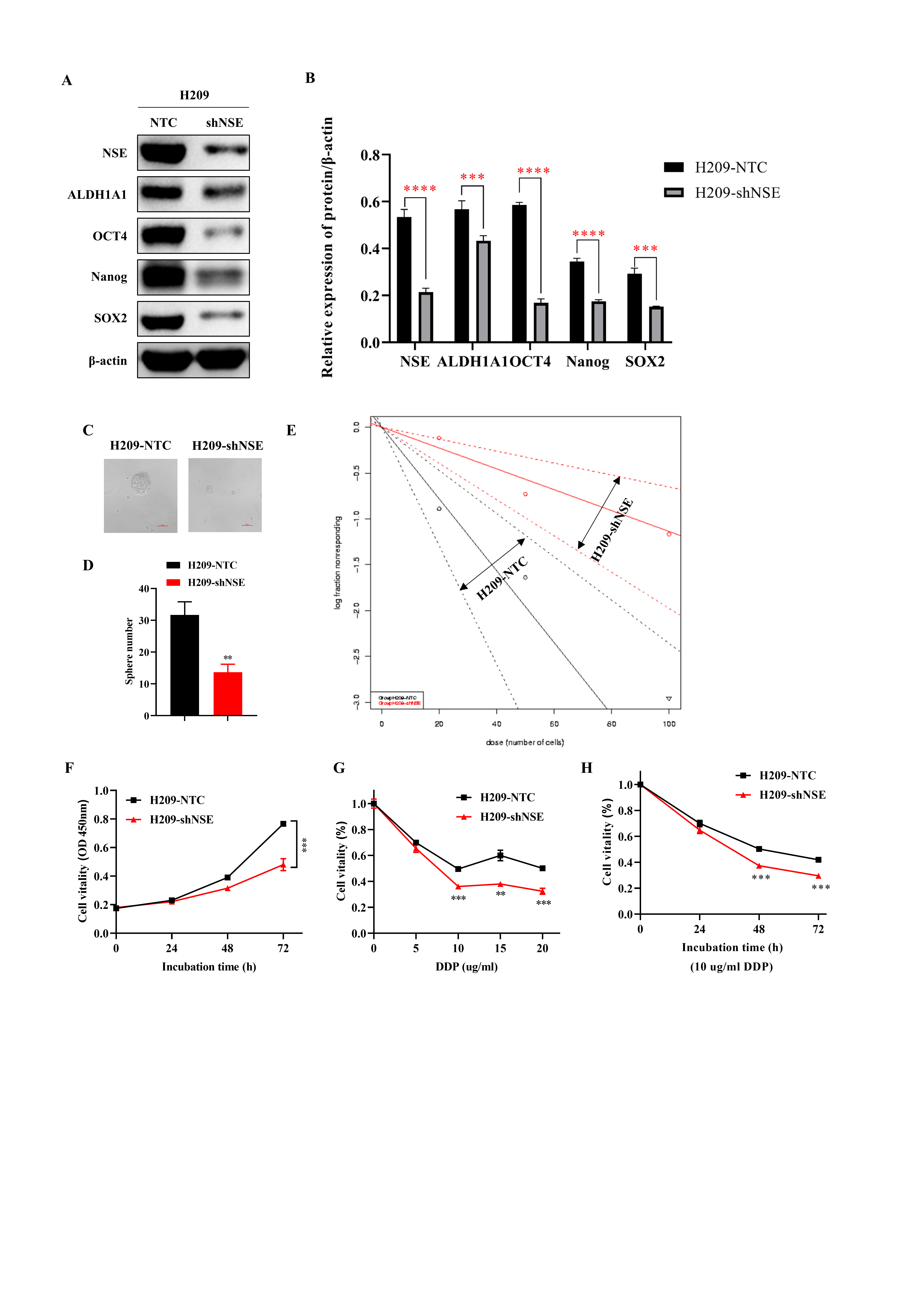

Supplement: Supplementary file 3 — Supplementary Figure 2 [file 41389_2022_396_MOESM3_ESM.tif]
